# A survey on the correlation between speech disorder and hemispheric lateralization

**Published:** 2012-11

**Authors:** Akram Soleimani, Habibollah Khazaee, Faranak Vakilifar

**Affiliations:** ^*a*^Kermanshah University of Medical Sciences, Kermanshah, Iran.; ^*b*^Department of Psychology, Kermanshah University of Medical Sciences, Kermanshah, Iran.; ^*c*^Bureau Chief of English language journal of JIVR, Deputy of Research and Technology, Kermanshah University of Medical Sciences, Kermanshah, Iran.

## Abstract

**Background:**

Speech and language, particularly at school time are among children’s main and sensitive issues. Thus, speech disorders can affect all personality and psychological aspects of an individual and make him/her facing lots of problems. One of the factors which may be effective in the onset of this impairment is handedness. Because of receiving reactions from their parents and environment, left handed children have more readiness to suffer from speech disorders. Therefore, the present study was aimed at investigating speech disorders and the relationship between the disorder and handedness.

**Methods:**

The community under study included primary school children in Kermanshah (Iran) and 600 students were selected through two-stage cluster sampling method proportional to the volume from three different regions of education and training. The samples were evaluated through phonetics test and continuous speech research in terms of pathological symptoms and the existence of symptoms of disorder by a speech and language specialist; and for the child’s left handedness or right handedness, he was asked to write a simple sentence proportionate with his educational grade. Finally, the data were collected, pathological symptoms were recorded in the related checklist and speech disorder was diagnosed.

**Results:**

A total of 600 children were participated in the study of them 39.5% were boys (mean age 9.12 ± 1.52 (SD)). 49 students (8.2 percent) were left handed. The rate of speech disorder was obtained 11.2% (67 students) of which 6 students were left handed. Prevalence of speech disorder for boys and girls was 11% and 11.3%, respectively.

Among the boys suffering the disorder, 7.7% was left handed, while this value for the girls was 9.8%. A significant correlation was not observed between the rate of disorder and gender and handedness (P less than 0.05).

**Conclusions:**

Although a significant statistical relation was not obtained between speech disorder and gender and handedness, the higher disorder prevalence among the female and left handed students can be attributed to the cultural context of male supremacy in Kermanshah and cultural and social pressures on women. These cases are considered as stress factors in increasing prevalence of the disorder. On the other side, the use of left hand is not so acceptable in various cultures; and in different societies, it may result in the child’s punishment. Furthermore, most of modern industrial tools have been mostly intended for the right handed; and these factors cause the left handed child to be under a constant pressure to change the handedness and the subsequent emotional excitements of speech disorders in him.

**Keywords:**

Speech disorder, Handedness, Phonetics test


Volume 4, Suppl. 1 Nov 2012
Publisher: Kermanshah University of Medical Sciences
URL: http://www.jivresearch.org
Copyright: (cc) BY

Paper No. 85

### A survey on the correlation between speech disorder and hemispheric lateralization

Akram Soleimani [a,*], Habibollah Khazaee [b], Faranak Vakilifar [c]

[a] Kermanshah University of Medical Sciences, Kermanshah, Iran.
[b] Department of Psychology, Kermanshah University of Medical Sciences, Kermanshah, Iran.
[c] Bureau chief of English language Journal of JIVR, Deputy of Research and Technology, Kermanshah University of Medical Sciences, Kermanshah, Iran.

### Abstract:

**Background:** Speech and language, particularly at school time are among children's main and sensitive issues. Thus, speech disorders can affect all personality and psychological aspects of an individual and make him/her facing lots of problems. One of the factors which may be effective in the onset of this impairment is handedness. Because of receiving reactions from their parents and environment, left handed children have more readiness to suffer from speech disorders. Therefore, the present study was aimed at investigating speech disorders and the relationship between the disorder and handedness.
**Methods:** The community under study included primary school children in Kermanshah (Iran) and 600 students were selected through two-stage cluster sampling method proportional to the volume from three different regions of education and training. The samples were evaluated through phonetics test and continuous speech research in terms of pathological symptoms and the existence of symptoms of disorder by a speech and language specialist; and for the child's left handedness or right handedness, he was asked to write a simple sentence proportionate with his educational grade. Finally, the data were collected, pathological symptoms were recorded in the related checklist and speech disorder was diagnosed.
**Results:** A total of 600 children were participated in the study of them 39.5% were boys (mean age 9.12 ± 1.52 (SD)). 49 students (8.2 percent) were left handed. The rate of speech disorder was obtained 11.2% (67 students) of which 6 students were left handed. Prevalence of speech disorder for boys and girls was 11% and 11.3%, respectively.
Among the boys suffering the disorder, 7.7% was left handed, while this value for the girls was 9.8%. A significant correlation was not observed between the rate of disorder and gender and handedness (P less than 0.05).
**Conclusions:** Although a significant statistical relation was not obtained between speech disorder and gender and handedness, the higher disorder prevalence among the female and left handed students can be attributed to the cultural context of male supremacy in Kermanshah and cultural and social pressures on women. These cases are considered as stress factors in increasing prevalence of the disorder. On the other side, the use of left hand is not so acceptable in various cultures; and in different societies, it may result in the child's punishment. Furthermore, most of modern industrial tools have been mostly intended for the right handed; and these factors cause the left handed child to be under a constant pressure to change the handedness and the subsequent emotional excitements of speech disorders in him.

### Keywords:

***Speech disorder, Handedness, Phonetics test***

* ***Corresponding Author at:***
***Akram Soleimani:*** *Deputy of Research and Technology, building No. 2, Kermanshah University of Medical Sciences, Shahid Beheshti Blv, Kermanshah, Iran. Phone: +98(918)-3366548, Email: akramsoleimani@gmail.com,* ***(Soleimani A.).***

